# Impact of resistance exercise program on muscle strength, cardiopulmonary function and glycolipid metabolism of bedridden population aged 80 years and above: A randomized controlled trial

**DOI:** 10.1097/MD.0000000000038412

**Published:** 2024-06-14

**Authors:** Yingjie Wang, Xiaopeng Huo, Xiaojing Wang, Hongwei Zhu, Xiaoxing Lai, Tong Yu

**Affiliations:** a Department of Health Care, Peking Union Medical College Hospital, Chinese Academy of Medical Sciences and Peking Union Medical College, Beijing, China; b Department of Nursing, Peking Union Medical College Hospital, Chinese Academy of Medical Sciences and Peking Union Medical College, Beijing, China.

**Keywords:** advanced age, cardiopulmonary function, glycolipid metabolism, muscle strength, resistance exercise

## Abstract

**Background:**

This study aimed to evaluate the impact of a resistance exercise program in the bedridden older adults in China.

**Methods:**

The patients aged 80 years and above with stable diseases were randomly divided into control group (receiving routine treatment and nursing) and training group (receiving the elastic ball and elastic band training applied for 55 minutes, 3 times a week during 6 months).

**Results:**

A total of 59 patients (control group: 30; training groups: 29) completed the study. In terms of muscle strength, the patients of the training group had better grip strength and supine leg lifts and 30-s sit-to-stand actions. In terms of cardiopulmonary function and glycolipid metabolism, the patients in the training groups had better lung capacity and high-density lipoprotein.

**Conclusion:**

The low-load and low-intensity resistance training may effectively improve not only the muscle strength of the bedridden older adults, but also the lung function and blood lipid metabolism.

## 1. Introduction

With the aggravation of China’s aging population, China has nearly 24 million of the older adults aged over 80 years^[1]^ and the number of bedridden older individuals is increasing with advancing age. In China, it has been reported that the proportion of people in the 60 to 69 age group, 80 to 84 age group, 90 to 94 age group and older adults over 100 who have limitations in mobility is 5%, 20%, 40%, and > 60%, respectively.^[2]^ The long-term bedridden condition in the older adults has serious consequences, especially in those who are aged 80 years and above. Moreover, once an older adult becomes bedridden, the possibility of rehabilitation is limited. Therefore, prevention and treatment of such conditions in the older adults is a critical medical need.^[3]^

Frailty and decrease in bone content and muscle strength are the important independent influencing factors in the bedridden older adults.^[4]^ According to the statistics, the muscle strength of people aged 80 years and above is less than half of that of healthy adults between the ages of 18 and 60.^[5]^ Studies have shown that skeletal muscle plays an important role in maintaining daily physical activities, balancing the blood lipid and triglyceride metabolism and preventing the occurrence of type 2 diabetes.^[6–8]^ Therefore, it is very important to improve the muscle quality and strength for a better health and quality of life in the older adults. Recently, resistance training for the older adults has gained more attention with studies establishing its benefits, and studies have confirmed that resistance training can prevent and treat osteoarthritis, diabetes, and blood lipid and heart diseases, improve muscle strength, reduce inflammatory reaction, and increase bone density.^[9–11]^ Therefore, strength training is extremely essential for the older adults.^[11]^

In 2014, the American Sports Medical Association published the design and demonstration of resistance training fitness program,^[12]^ including healthy older adults, or older adults with certain diseases, such as diabetes, dementia, hypertension, osteoporosis, dysfunctional mobility or disability, and the fitness effect has been well verified theoretically and empirically. The theoretical research of resistance training mainly includes: the necessity and strength of strength training for the older adults, adaptive changes caused by training, such as changes in muscle strength, protein and hormones after resistance training, effects on diseases, such as diabetes, cardiovascular disease and bone metabolism.^[13,14]^ The empirical research mainly includes: the effects of strength training or resistance training on muscle strength, hormones, inflammatory factors, vascular endothelial function, blood lipid and blood pressure in the older adults.^[15,16]^ The recent study on strength training in the older adults have several limitations.^[1]^ For instance, Xu et al^[17]^ developed an integrated system for monitoring muscle state, which requires specialized instruments.Additionally, Smeuninx et al^[18]^ evaluated the effect of short-term resistance exercise training on older men. Although it yielded positive outcomes, the small number of patients involved limits its broader application. Moreover, most of the fitness programs included in these studies used a combination of exercise training, or mainly the aerobic exercise. In China, bedridden older adults are mostly hospitalized or stay at home. They are not financially well-off, which makes the training program unsuitable for them.^[2]^ The majority of participants in these studies were older adults capable of self-care, while those with movement disorders or disabilities were fewer in number.

Therefore, the aim of this study is to develop a resistance exercise program suitable for the bedridden older adults in China, and explore its impact on the cardiopulmonary function, muscle strength and glycolipid metabolism of such patients.

## 2. Materials and methods

### 2.1. Study design and participants

In this randomized controlled trial, the patients aged 80 years old and above with stable diseases, who were hospitalized in the geriatric ward, were consecutively enrolled at Peking Union Medical College Hospital from March 2020 to April 2020.

Inclusion criteria: patients in whom the disease such as diabetes, hypertension, dysfunctional mobility, chronic heart disease, pneumonia, chronic obstructive pulmonary disease, osteoporosis or disability and so on was in a stable stage (symptoms were stable or mild, and related abnormal indicators were stable); patients aged 80 years and above; patients who were bedridden (those whose activities to maintain basic physiological needs, such as diet, excretion, must be carried out in bed, except for standing beside the bed or taking a wheelchair for examination and treatment)^[19]^ for 1 month or more; patients in whom the ability to perform daily activities decreased due to the decline in muscle strength, such as long-term bed rest, sitting on a chair, or inability to go out alone, but can only perform indoor activities; however, they could perform a part of self-motion using their own strength in the lying posture, such as turning over, moving, leg lifts, knee standing, and other activities; patients with clear consciousness and can cooperate with sports.

Exclusion criteria: patients in whom New York Heart Association cardiac function was classified as grade III and IV; patients with severe liver and renal insufficiency; patients with malignant tumor; patients with severe infection; patients with left ventricular ejection fraction (LVEF) < 40%; and patients suffering from disorders of bone and joint, muscle, and nervous system and other diseases such as gout, osteoarthropathy and poliomyelitis and so on, which make them unable to cooperate with exercise assessment and treatment. Written informed consent was obtained from each participant. This study has been registered in China Clinical Trial Registration Center (ChiCTR2000031099) and approved by the ethics review committee of Peking Union Medical College Hospital, Chinese Academy of Medical Sciences (JS-2265).

### 2.2. Randomization, allocation and blinding

**Figure 1.**
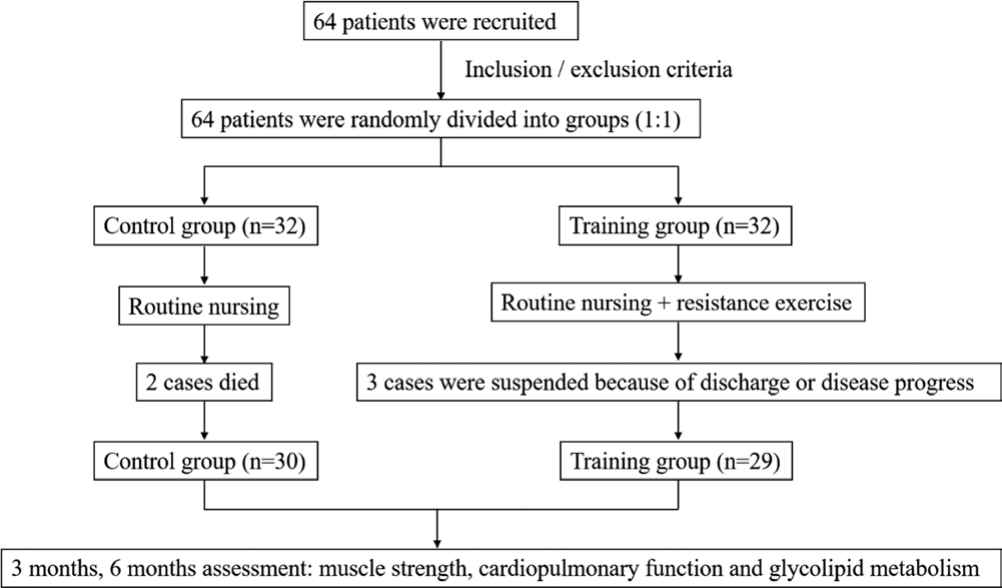
Flowchart of the study.

Firstly, the subjects were selected after hospitalization. And then, informed consent was signed by the patients and their families when joining the research project. The patients were randomly assigned to control and training groups according to the random number table with a ratio of 1:1 and the specific random methods were as follows: Research staffs drew up the number of the research objects first, used the random number table method to generate random numbers, and opened the next sequentially numbered opaque envelope that contained the product assignment. An individual unassociated with the clinical portion of the study prepared the envelopes. The subjects with odd random numbers were divided into training group and even number into control group. Single blind method was used and the subjects did not know their grouping and all intervention schemes of 2 groups. The flowchart of this study is shown in Figure 1.

### 2.3. Sample size estimation

The sample size was estimated according to the estimation formula for mean comparison of 2 independent samples: N1 = N2 = 2*[(t_α/2_ + t_β_)*s/δ]^2^. According to the research and calculation by Kui Zou et al,^[20]^ δ = 6.9 based on the grip strength, and the larger standard deviation of one of the 2 samples was considered as s = 8.2. In addition, bilateral α = 0.05, β = 0.1, and t_α/2_ = 1.96, and t_β_ = 1.282 based on the statistic table. By adding these values into the abovementioned formula, the number of samples required in each group was estimated to be 29. As the rate of loss of follow-up was considered to as 10%, 32 patients were finally considered in each group, and the total sample size was 64 patients.

### 2.4. Intervention

In this study, the medical staff gave routine treatment and nursing care, including the education on bed activities to the patients of the control group in the ward. For example, the guidance and training on some of the necessary daily activities that the patients will have to perform being bedridden, such as eating, dressing, grooming, and defecation, were provided every day. In addition, the patients were assisted by the medical staff in every 2 hours to turn over in the bed, clap on the back, or change the body position. The local skin massage of patients was performed regularly.

The Tenth Edition of the American College of Sports Medicine’s Guidelines for Exercise Testing and Prescription, authored by the American College of Sports Medicine, recommends a specific regimen for elastic band training aimed at middle-aged and elderly populations. Following this guideline, our study’s exercise program was meticulously designed to specify load intensity, training volume, frequency, rest periods, and the sequence of exercises. To refine our program, we employed the Delphi method, engaging in 2 rounds of consultations with a panel of 15 specialists in geriatric clinical nursing, geriatric medicine, and exercise rehabilitation. Considering the advanced age of our study participants, who are 80 years old and above, the panel unanimously chose a blend of low-intensity, low-load exercises using elastic balls and bands. The consultation yielded high engagement, with response rates of 93.33% and 100% for the 2 rounds, respectively. The analysis of expert agreement produced a coordination coefficient of 0.772 with a *P* value less than .05, and Cronbach’s alpha values of 0.713 and 0.792 for each round, confirming the program’s reliability and authority.

**Figure 2.**
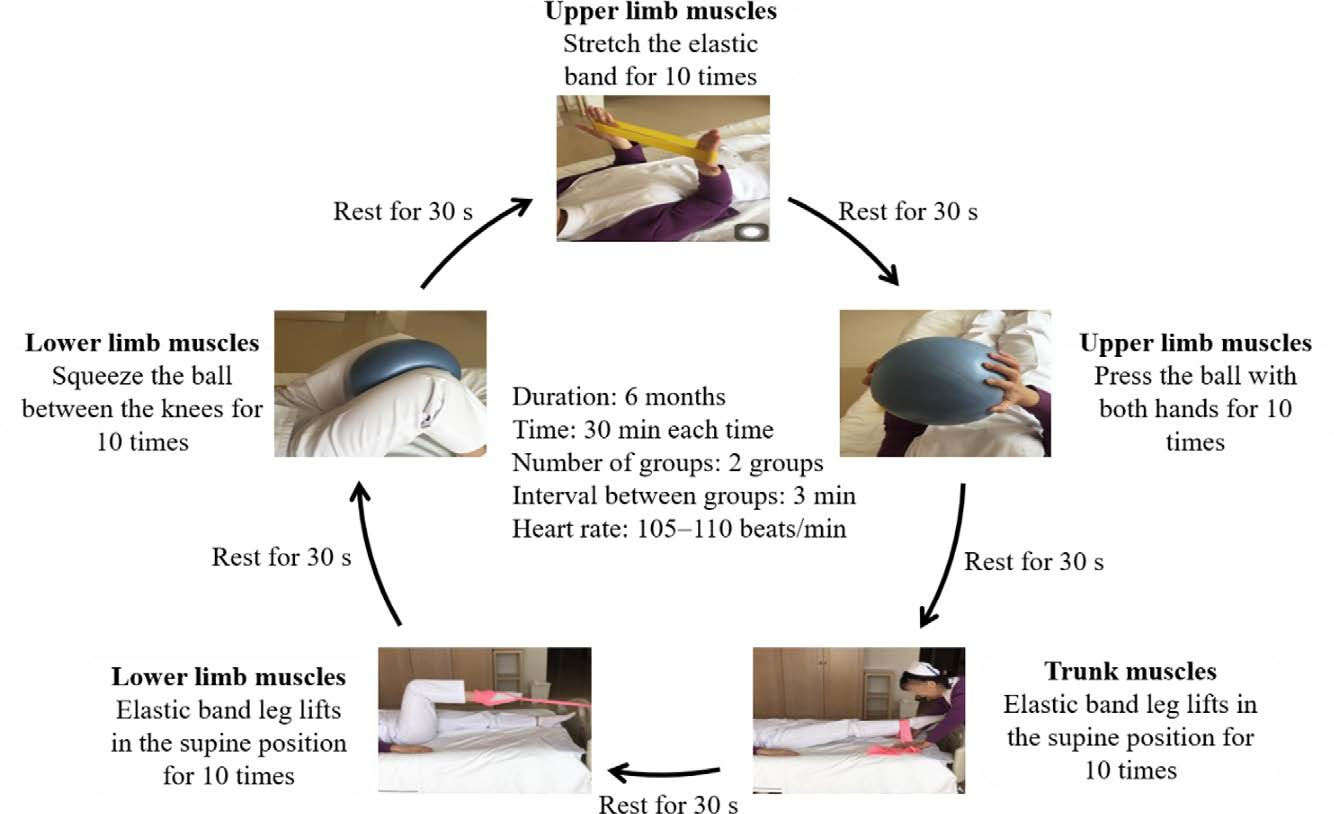
Supine resistance exercise program.

The patients in the training groups were guided by the research team for a 6-month low-load resistance training, including warm-up preparation stage, basic training stage, and finishing and relaxation stage. The total duration of exercise was 55 minutes, including10 minutes of warm-up activities, 35 minutes of resistance training, and 10 minutes of finishing and relaxation. Warm-up and finishing and relaxation activities included a massage from the head to the ankle, maintaining the average heart rate at about 100 beats/min. The basic training stage included strength training actions for the upper limb muscles (horizontal stretching of an elastic band + pressing of an elastic ball with both hands), the trunk muscles (elastic band leg lifts in the supine position), and the lower limb muscles (elastic band leg lifts in the supine position + squeezing the ball between the knees; Fig. 2). Exercise for 2 cycles per training. Each action was repeated 10 times and the interval between each action was 30 seconds. The interval time between the training cycles was 3 minutes, which was adjusted according to the physical condition of the older adults, such as appropriately extending the interval time when feeling tired. The type of training was low load with low intensity (i.e., 30–50% of maximum load), and the average heart rate was controlled at 105 to 110 beats/min (Table 1). Exercise guidelines issued by the American Heart Association and the American Sports Medical Association suggest that the exercise intensity of the older adults should be based on the rating of perceived exercise (RPE) scale.^[21]^ The relationship between heart rate and RPE is about RPE value × 10 = heart rate, and level 10 to 11 (relaxed) of RPE (Brog6-20) scale is low intensity (30–50% of maximum load).^[22]^ Therefore, this project monitors the exercise intensity through the heart rate and RPE scale. Continuous electrocardiogram (ECG) monitoring should be conducted in patients during the exercise process, and attention should be paid to control the rhythm. The patients’ subjective feelings should be asked according to the RPE scale and the intensity should be adjusted to level 10 to 11 during the training. The training was conducted every Monday, Wednesday, and Friday (3 times a week) for a total of 6 months. The elastic ball used in this study was a Pilates ball, with a diameter of 20 cm, and the elastic band used was the lowest resistance band (red elastic band).

**Table 1:** Intensity and amount of resistance exercise.

Exercise action	Duration (months)	Number of cycles	Time (min)	Interval between each time (s)	Interval between groups (min)	Color of elastic band or diameter of ball (cm)
Horizontal stretch of elastic band	6	2	3	30	3	Red
Pressing ball with both hands	6	2	3	30	3	20
Elastic band leg lifts in the supine position	6	2	3	30	3	Red
Elastic band leg lifts in the supine position	6	2	3	30	3	Red
Squeezing the ball between the knees	6	2	3	30	3	20

For a conventional and complete intervention, the research team is comprised of 2 postgraduate students, one physiotherapist, one geriatric specialist, one geriatric specialist nurse, and one rehabilitation specialist nurse. Each member holds at least a bachelor’s degree and brings over 5 years of professional experience, equipping them to provide project guidance and follow-up. The graduate students, physiotherapist, and geriatric specialists primarily focus on designing exercise training programs through interviews and discussions grounded in theoretical knowledge. The physiotherapist, along with rehabilitation physicians, offers standardized guidance and training on exercise routines and the use of assistive devices to the intervention team, which includes one specialist nurse, 8 lead nurses, and patient caregivers. This training was delivered in 2 sessions, each lasting 2 hours, with all participants successfully completing an assessment afterwards. For the intervention phase, one specialist nurse, 8 lead nurses, and the patients’ personal caregivers were actively involved in the exercise sessions. The lead nurses were organized into 4 teams, with 2 nurses per team, where one nurse provided direct one-on-one exercise guidance to a patient, and the other monitored the patient’s ECG to prevent high heart rates and manage subjective fatigue levels. Each patient was continuously accompanied by a nurse or caregiver, ensuring one-to-one care and safety measures, including bed protection. A specialist nurse conducted regular follow-ups to oversee the quality of the intervention, compiled feedback on outcomes, and reported to the research team, which then adjusted the intervention strategies as needed based on this feedback. The effectiveness of these interventions was evaluated by the 2 graduate students through face-to-face assessments at 3 to 6 months post-intervention.

Indeed, during the training period, patients may be discharged due to improvements in their health. Following the initiation of training, regular treatment and medication were administered as required by the patient’s condition, alongside a diet rich in proteins and calories tailored to their energy needs, complemented by dietary advice. The training took place within the hospital ward for the elderly during their stay and continued at home post-discharge. The process was as follows: While hospitalized, the ward’s lead nurse conducted regular interventions for the patients, lasting at least 1 month, to ensure they learned the training techniques and skills effectively. Patients were supervised one-on-one by nurses or caregivers, with specialist nurses conducting weekly follow-ups to monitor and ensure the intervention’s success. Prior to discharge, caregivers received additional training to fully grasp all exercise techniques, maintaining one-on-one supervision during workouts to guarantee intervention continuity. A follow-up schedule was set up, inviting patients to join a resistance training group via QR code, and distributing exercise aids like elastic bands and balls. Post-discharge, caregivers uploaded weekly training videos for the research team’s review. Monitoring and evaluation of the training’s execution were conducted through phone calls, online check-ins, or scheduled visits to address any deviations or potential risks promptly. Additionally, emergency protocols for incidents such as falls, difficulty breathing, or chest pain were established and rehearsed. In any such events, immediate medical help was sought, with the research team responding according to the emergency plans. Moreover, the team provided ongoing education and encouragement through various follow-up methods, regularly shared updates and feedback on the training progress, and made necessary adjustments to the intervention. This ensured that patients could successfully complete the 6-month exercise regimen.

### 2.5. Compliance and security

Compliance is determined by investigating whether the subject has completed all exercises as required. The safety of the training is determined by investigating whether the study subjects have sports-related injuries such as joint muscle damage, cardiovascular or cerebrovascular damage. Certain compliance strategies were strictly followed for the safety of the patients. First, during every training process, it is necessary to ensure that 1 patient was protected by a professional medical staff and an accompanying staff to ensure his or her safety at the bedside. Second, the guidance on correct breathing mode: exhalation during the lift or finish phase and inhalation during the lower or return to start position. Third, avoidance of high heart rate: continuous ECG monitoring should be conducted during the exercise process, and attention should be paid to slow down the rhythm. After each action, patients were guided to rest for 30 seconds to avoid high heart rate. All the nurses should have been guided and trained by rehabilitation physiotherapists, and have 5 years or more of experience in geriatric nursing. Moreover, they have rich experience in sports rehabilitation nursing to ensure the consistency in operation methods and strength, reducing mixed factors.

### 2.6. Muscle strength, cardiopulmonary function and glycolipid metabolism assessment

In this study, the muscle strength (primary indicators), cardiopulmonary function and glycolipid metabolism (secondary indicators) of the patients were evaluated in a face-to-face manner before intervention and at the third and sixth months after intervention.

### 2.7. Muscle strength evaluation

#### 2.7.1. Grip strength

Electronic hand dynamometer (EH101; CAMRY, Xiangshan Weighing Apparatus Group Co., Ltd, Guangdong, China) was used to evaluate the muscle strength of the upper limbs. The upper and lower handles of the device were tightly held by the powerful hand of the patient with maximum strength for 2 seconds. The test was performed twice and the maximum value was used for analysis.^[21,23]^

#### 2.7.2. Leg lifts in the supine position

This action was used to evaluate the strength of the trunk muscles. After the patients lay flat on the bed, a double column pole, connected with a rubber band at the height of 40 cm, was placed on his or her each side. Then asked the patients to do the abdominal straight leg lifting, and put down their feet slowly after touching the rubber band. The number that the patients completed this action within 1 minute was recorded for analysis.

#### 2.7.3. Repeated sit-to-stand action for 30 seconds

This action was used to evaluate the strength of lower limb muscles (for patients who could get out of the bed, the number was calculated as true; for patients who could not get out of bed, the number was 0). The patients sat on a chair (with a seat height of 43 cm), back straight and hands folded across the chest. Then, they had to get up from the chair to a standing position and sit back down on the chair. These actions were repeated for 30 seconds. The total number of actions within 30 seconds was recorded.

The stronger grip strength or the more times of leg lifts and sit-to-stand actions of patients indicated that they muscle strength was better. Unified indicators were collected by trained research team using unified measurement and observation tools.

### 2.8. Cardiopulmonary function and glycolipid metabolism assessment

According to the standard equipment and testing standards stipulated by national physique monitoring,^[24]^ the blood pressure of participants in this study were measured using a blood pressure meter (XJ11D; Shanghai Medical Equipment Co., Ltd, Shanghai, China), resting heart rate (RHR) was measured using finger clip pulse oximeter (Tuffsat; GE Healthcare Finland Oy, Madison), LVEF% was measured by cardiac color Doppler ultrasound (portable color ultrasound diagnostic system, model: PHILIPS CX50; Philips Ultrasound, Bothell) and vital capacity was measured using a vital capacity tester (NL-100; YiwuQiyue Sporting Goods Co., Ltd, Yiwu, Zhejiang, China). The RHR, blood pressure, LVEF% were measured when the patients lied flat at rest. The normal range of heart rate is 60 to 100 beats/min, and high blood pressure is 90 to 140 mm Hg and low pressure is 60 to 90 mm Hg (1 mm Hg = 0.133 kpa). The normal range of left ventricular ejection fraction is 50% to 70%. The vital capacity was measured when the patients were in sitting position and the normal range is 2500 to 3500 mL for women and 3500 to 4000 mL for men.

The ward specialist nurse collected 8 mL of venous blood from all participants under fasting conditions in the morning. The levels of blood glucose, high-density lipoprotein (HDL), low-density lipoprotein (LDL), total cholesterol, and triglyceride were detected by the hospital laboratory center. The closer the index value is to the standard range, the better the cardiopulmonary function and glycolipid metabolism is.

### 2.9. Conditions for stopping training during intervention

In this study, the training should be stopped immediately if adverse conditions occurred in patients, such as asthma, dyspnea, high heart rate, pain, or muscle weakness.

### 2.10. End point and follow-up

The endpoint of this study is the improvement of the patient’s muscle strength, cardiopulmonary function and glycolipid metabolism. In this study, the patients were followed up until the end of the study, and the muscle strength, cardiopulmonary function and glycolipid metabolism indexes of the patients were tested in the third and sixth months of the study.

### 2.11. Statistical analysis

EpiData3.1 (EpiData Association, Odense, Denmark) was used for data entry, and SPSS23.0 statistical analysis software (IBM Corp, Armonk) was used for analysis based on the Per Protocol Set (PP set) which included the subjects carrying out the test in strict accordance with the scheme, and completing the whole process of scheme design. The continuous data with normal distribution were represented as mean ± standard deviation and those with non-normal distribution as median (Q25, Q75). For the continuous data with normal distribution, the independent two-sample *t* test or paired two-sample *t* test was used for comparison between groups, and repeated measurement analysis of variance was used for intra-group comparisons. The continuous data with non-normal distribution were analyzed using the Mann–Whitney *U* test, the Wilcoxon signed rank sum test, and the Friedman test. Categorical data were represented as count (percentage) and were analyzed using Fisher’s exact probability and χ^2^ tests. *P* < .05 was considered as statistically significant.

## 3. Results

### 3.1. General information

64 patients were enrolled in this study. Among them, 2 patients died of pulmonary infection in the control group. In the training groups, 2 patients who discharged from the hospital and 1 patient who was treated with ventilator due to aspiration pneumonia discontinued the training process. Finally, 59 patients (30 patients in the control group and 29 patients in the training groups) completed the 6-month training process and evaluation. Of these, 39 patients were male (66.10%) and 20 patients female (33.90%), with an average age of 95.73 ± 4.60 years (82–104 years). The average duration of staying in bed was 9.34 ± 5.14 years. 55.20% of participants had one disease, 25.40% 2 diseases, 7 subjects 3 or more diseases and only 5 subjects were not ill. The types of diseases suffered by the patients (from high to low) were pneumonia, hypertension, and diabetes; of which, 71.20% patients had a surgical history, mainly cataract surgery, tumor resection, and cholecystectomy. No significant differences were observed in the general information of patients between the training and control groups (*P* > .05) as shown in Table 2.

**Table 2:** Baseline characteristics of patients.

Characteristics	Total (n = 59) (%)	Control group (n = 30) (%)	Training groups (n = 29) (%)	*P*
Age (yr)	95.73 ± 4.60	95.40 ± 4.21	96.07 ± 5.02	.581
Gender				
Male	39 (66.10)	22 (73.30)	17 (58.60)	.233
Female	20 (33.90)	8 (26.70)	12 (41.40)	
Body weight (kg)				
N/A because of not getting out of bed at all (n = 20)	20 (33.90)	11 (36.70)	9 (31.00)	.244
Other (those who can stand beside the bed or Wheelchair or perform indoor activities only) (n = 39)	60.74 ± 11.40	60.06 ± 11.83	61.45 ± 11.10	.708
Bed time (all participants’ length of stay in bed) (yr)	9.34 ± 5.14	9.97 ± 5.11	8.73 ± 5.16	.334
Kinds of diseases (numbers)				
0	5 (8.50)	1 (3.30)	4 (13.80)	.177
1	32 (54.20)	14 (46.70)	18 (62.10)	
2	15 (25.40)	10 (33.30)	5 (17.20)	
≥3	7 (11.90)	5 (16.70)	2 (6.90)	
Diagnosis				
Pneumonia	16 (27.10)	9 (30.00)	7 (24.10)	.235
Hypertension, diabetes	10 (16.90)	4 (13.30)	6 (20.70)	
Fever	5 (8.50)	1 (3.30)	4 (13.80)	
Gallstones and cholecystocele	4 (6.80)	2 (6.70)	2 (6.90)	
Cerebral infarction	3 (5.10)	2 (6.70)	1 (3.40)	
Chronic obstructive pulmonary disease	3 (5.10)	1 (3.30)	2 (6.90)	
Lung cancer	3 (5.10)	1 (3.30)	2 (6.90)	
Other	15 (25.40)	10 (33.40)	5 (17.30)	
History of surgery				
Yes	42 (71.20)	22 (73.30)	20 (69.0)	.711
No	17 (28.80)	8 (26.70)	9 (31.0)	

Continuous variables were expressed as mean ± standard deviation, and classified variables were expressed as frequency (percentage). *P* value was determined from the comparison between control and training groups.

### 3.2. Muscle strength

**Figure 3.**
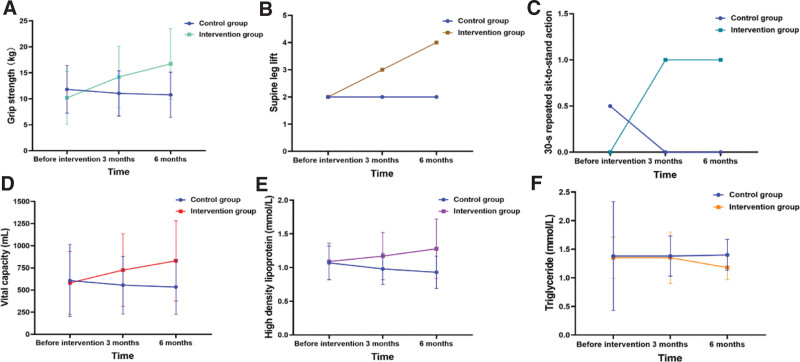
Comparison of (A) Grip strength, (B) Supine leg lift, (C) 30-s repeated sit-to-stand action, (D) Vital capacity, (E) HDL, and (F) Triglyceride between the 2 groups with time extension.

Before training, no significant differences were observed in the grip strength, number of leg lifts in the supine position, and number of 30-s repeated sit-to-stand actions between the 2 groups (*P* > .05). After training, there is an interaction between the grip strength training and the training time (*P* < .001; Table 3). In 3 and 6 months after training, the upper-limb grip strength of patients in the training groups was better than that of the control group (*P* = .024; *P* < .001; Table 3). In the control group, the upper-limb grip strength of patients in 3 and 6 months all decreased than before (*P* = .001; *P* < .001), while in the training groups all increased than before as time prolonged (*P* < .001; Table 3; Fig. 3A).

**Table 3:** Comparison of muscle strength between the 2 groups before and after intervention.

Indicator	Control group (n = 30)	Training groups (n = 29)	*P*
Grip strength (upper limb muscle strength, kg)*			
Before intervention	11.81 ± 4.59	10.19 ± 5.09	.204
At 3 months	11.04 ± 4.36†	14.19 ± 5.90†	.024
At 6 months	10.76 ± 4.35†	16.72 ± 6.76†^,^‡	<.001
Supine leg lift (trunk muscle strength, times)			
Before intervention	2 (0, 6.50)	2 (0, 6)	.863
At 3 months	2 (0,5.75)	3 (2, 8.5)†	.060
At 6 months	2 (0, 5.25)†	4 (2, 9)†^,^‡	.010
30-s repeated sit-to-stand action (lower limb muscle strength, times)			
Before intervention	0.5 (0, 2)	0 (0, 2.5)	.823
0 times (n, %)	15 (50.00)	17 (58.62)	
1–3 times (n, %)	10 (33.33)	8 (27.59)	
4–6 times (n, %)	5 (16.67)	4 (13.79)	
At 3 months	0 (0, 2)	1 (0, 3)	.243
0 times (n, %)	16 (53.33)	13 (44.83)	
1–3 times (n, %)	10 (33.33)	11 (37.93)	
4–6 times (n, %)	4 (13.34)	5 (17.24)	
At 6 months	0 (0, 1)†	1 (0, 4)†	.032
0 times (n, %)	17 (56.67)	12 (41.38)	
1–3 times (n, %)	13 (43.33)	8 (27.59)	
4–6 times (n, %)	0	7 (24.14)	
≥7 times (n, %)	0	2 (6.89)	

The continuous variables in normal distribution were expressed as mean ± standard deviation. The continuous variables in non-normal distribution were expressed as median (Q25, Q75).

* *F*_time_ = 28.241, *P*_time_ < .001, *F*_group_ = 3.614, *P*_group_ = .062, *F*_interaction_ = 50.139, *P*_interaction_ < .001.

† *P* < .05 vs before intervention.

‡ *P* < .05 vs 3 months.

At 6 months after training, leg lifts in the supine position and 30-s repeated sit-to-stand actions of patients in the training groups were better than those in the control group (leg lifts in the supine position, *P* = .010 and 30-s repeated sit-to-stand action, *P* = .032; Table 3). In the control group, the abilities to perform leg lifts in the supine position (*P* = .028) and 30-s repeated sit-to-stand actions (*P* = .029) decreased at 6 months compared with before, with the statistical significance. In the training groups, the ability to perform leg lifts in the supine position increased at the third month compared with before (*P* = .002), and it was better at 6 months than that at 3 months (*P* = .009) and before (*P* < .001). The ability to perform 30-s repeated sit-to-stand actions of the patients in the training groups at 6 months was better compared with before (*P* = .001), and the differences were statistically significant (Table 3; Fig. 3B and C).

### 3.3. Cardiopulmonary function

In this study, no significant difference was observed in the blood pressure, heart rate, left ventricular ejection fraction, and vital capacity compared with the levels in patients of both groups before training (*P* > .05; Table 4). After training, there was an interaction between the training time and training of vital capacity (*P* < .001). The vital capacity was significantly better in the training group than that in the control group at 6 months (*P* = .005; Table 4). In the control group, the vital capacity decreased at 6 months compared with 3 months and before, with the statistical significance (*P* = .010; *P* = .044), and in the training group, it increased with the lapse of time and the differences were all statistically significant (*P* < .001; Table 4; Fig. 3D).

**Table 4:** Comparison of cardiopulmonary function and glycolipid metabolism between the 2 groups before and after intervention.

Indicators	Control group (n = 30)	Training groups (n = 29)	*P*
Blood pressure (mm Hg)*			
Before intervention			
Systolic blood pressure	134.30 ± 18.58	125.79 ± 19.42	.091
Diastolic blood pressure	64.27 ± 10.88	60.31 ± 9.57	.144
At 3 months			
Systolic blood pressure	127.77 ± 19.52	128.21 ± 8.99	.911
Diastolic blood pressure	63.57 ± 8.44	63.28 ± 8.66	.897
At 6 months			
Systolic blood pressure	134.33 ± 15.42	127.10 ± 14.72	.071
Diastolic blood pressure	64.33 ± 10.83	60.93 ± 8.51	.186
Heart rate (beats/min)			
Before intervention	78 (75.5, 82.25)	74 (69, 80.5)	.429
At 3 months	78 (68, 80)	77 (70, 80)	.891
At 6 months	74 (67.75, 80.5)	75 (70, 79.5)	.921
Left ventricular ejection fraction (%)†			
Before intervention	64.73 ± 6.53	64.69 ± 3.24	.974
At 3 months	64.90 ± 6.27	65.31 ± 3.20	.754
At 6 months	65.10 ± 6.94	66.13 ± 3.47#	.473
Vital capacity (mL)‡			
Before intervention	607.10 ± 407.63	580.69 ± 354.34	.792
At 3 months	555.27 ± 325.64	725.21 ± 410.61#	.083
At 6 months	533.73 ± 305.59#**	829.28 ± 452.27#**	.005
Blood glucose (mmol/L)			
Before intervention	6.25 (5.75, 8.63)	6.20 (5.25, 7.75)	.355
At 3 months	6.35 (5.30, 8.40)	5.70 (5.11, 7.20)	.160
At 6 months	5.90 (5.30, 7.95)	5.60 (5.25, 5.85)	.084
High-density lipoprotein (mmol/L)§			
Before intervention	1.07 ± 0.25	1.09 ± 0.27	.768
At 3 months	0.98 ± 0.23	1.17 ± 0.35	.018
At 6 months	0.93 ± 0.24#	1.28 ± 0.44#	.001
Low-density lipoprotein (mmol/L)‖			
Before intervention	2.04 ± 0.89	2.29 ± 0.75	.246
At 3 months	2.15 ± 0.86	2.18 ± 0.79	.881
At 6 months	1.90 ± 0.74	2.12 ± 0.61	.202
Total cholesterol (mmol/L)¶			
Before intervention	3.50 ± 0.95	3.65 ± 0.90	.552
At 3 months	3.52 ± 1.05	3.56 ± 0.87	.875
At 6 months	3.20 ± 0.85	3.54 ± 0.76	.117
Triglyceride (mmol/L)			
Before intervention	1.38 (0.89, 2.84)	1.35 (1.06, 1.74)	.529
At 3 months	1.38 (0.95, 2.46)	1.35 (0.89, 1.70)	.383
At 6 months	1.40 (0.82, 2.12)	1.18 (1.01, 1.51)#**	.396

The continuous variables in normal distribution were expressed as mean ± standard deviation. The continuous variables in non-normal distribution were expressed as median (Q25, Q75).

* Systolic blood pressure *F*_time_ = 0.971, *P*_time_ = .382, *F*_group_ = 1.998, *P*_group_ = .163, *F*_interaction_ = 2.805, *P*_interaction_ = .065, Diastolic blood pressure *F*_time_ = 0.371, *P*_time_ = .691, *F*_group_ = 1.731, *P*_group_ = .194, *F*_interaction_ = 1.073, *P*_interaction_ = .345.

† *F*_time_ = 3.818, *P*_time_ = .025, *F*_group_ = 0.128, *P*_group_ = .722, *F*_interaction_ = 1.360, *P*_interaction_ = .261.

‡ *F*_time_ = 14.941, *P*_time_ < .001, *F*_group_ = 2.276, *P*_group_ = .137, *F*_interaction_ = 37.623, *P*_interaction_ < .001.

§ *F*_time_ = 0.501, *P*_time_ = .581, *F*_group_ = 7.331, *P*_group_ = .009, *F*_interaction_ = 10.342, *P*_interaction_ < .001.

‖ *F*_time_ = 2.532, *P*_time_ = .092, *F*_group_ = 0.886, *P*_group_ = .350, *F*_interaction_ = 1.147, *P*_interaction_ = .316.

¶ *F*_time_ = 2.698, *P*_time_ = .072, *F*_group_ = 0.612, *P*_group_ = .437, *F*_interaction_ = 1.215, *P*_interaction_ = .301.

# *P* < .05 vs before intervention.

** *P* < .05 vs 3 months.

The training time of systolic blood pressure, diastolic blood pressure, and left ventricular ejection fraction had no interaction with the training (*P* = .065; *P* = .345; *P* = .261). The left ventricular ejection fraction of patients in the training group at 6 months was better than pre-intervention and the difference was statistically significant (*P* < .001), but there was no difference between 2 groups at 3 and 6 months (*P* > .05; Table 4). There was no statistical significance in the inter- and intra-group comparison of other indicators (*P* > .05). Similarly, the difference in intra- and inter-group heart rate was not statistically significant (*P* > .05).

### 3.4. Glycolipid metabolism

Before training, there was no statistically significant difference in the blood glucose, HDL, LDL, total cholesterol, or triglycerides between the 2 groups (*P* > .05). After training, there was no interaction between the training time and training of LDL and total cholesterol (*P* = .318; *P* = .240), and there was no statistically significant difference between and within groups (*P* > .05). Furthermore, there were no significant inter- or intra-group differences in blood glucose (*P* > .05). There was a significant interaction between the HDL training time and the training (*P* < .001). The HDL of patients in the training group was better than that of patients in the control group at 3 and 6 months, and the difference in both cases was statistically significant (*P* = .018; *P* = .001; Table 4). In the control group, the HDL of patients at 6 mouths was getting worse than pre-intervention, while in the training group, it was better than before, and the differences were all statistically significant (*P* = .048; *P* = .017; Table 4; Fig. 3E). There was no significant difference in the triglyceride levels of patients between the 2 groups at each time point (*P* > .05; Table 4). In the control group, there was no significant difference in the triglyceride levels of the patients before and after training (*P* = .150). But in the training group at 6 months, the triglyceride level of patients was significantly lower than that at the baseline and at 3 months after training (*P* = .002; *P* = .038; Table 4; Fig. 3F).

### 3.5. Compliance and security

During the entire study process, no patient had any related safety events such as joint muscle damage, cardiovascular, or cerebrovascular damage. Two patients died of pulmonary infection in the control group and 3 patients discontinued the intervention process in the training groups (2 patients discharged from the hospital and 1 patient was treated with ventilator due to aspiration pneumonia). Under the meticulous care of the nursing staff, the remaining patients completed the 6-month training process and evaluation, with a compliance of 92.19%.

## 4. Discussion

In this study, a low-load and low-intensity resistance training for 55 minutes with the elastic ball and elastic band conducted, 3 times in a week during 6 months was used in older adults over the age of 80 years. The results showed that the program was simple, safe, effective, and no intolerance or other adverse reactions occurred.

The older adults had high compliance (92.19%) and good feedback about the training program. This was similar with Idland G’s research, who applied a 12-week program of progressive resistance exercise on a group of nonagenarian (≥90 years) community-dwelling women, and the results showed that progressive resistance training was a safe and efficient method to enhance mobility and increase lower body strength.^[25]^ The American Heart Association and the American College of Sports Medicine jointly issued a Fitness Guideline, pointing out that the older adults aged over 65 years should have 8 to 10 different kinds of resistance training, such as dumbbell, elastic band exercise, roller skates and binding sandbag, for at least 2 to 3 times a week^[26]^ The age of intervention population in these studies ranged from 60 to 90 years, including not only the healthy older adults, but also those with underlying diseases, such as diabetes,^[27]^ dementia,^[28]^ hypertension,^[29]^ osteoporosis,^[30]^ mobility disorder or disability,^[31]^ and the training effect was also relatively comprehensive including long-term body changes and short-term changes within 30 minutes such as blood glucose, blood pressure and heart rate, etc.^[32]^ In China, aerobic exercise is considered the major method of exercise for the older adults; whereas, resistance exercise has mainly focused on theoretical researches but less practical studies with scientific guidance.^[33,34]^ The resistance exercise with the elastic ball and elastic band used in this study is a kind of training method to overcome resistance to complete muscle contraction and enhance motor functions of the muscles, joints, and ligaments along with the change in body shape of patients. It is the most commonly used circulation resistance exercise method, which not only improves the cardiovascular function and muscle endurance, but also increases the muscle strength.^[11]^ The expert unanimously points out that the elastic band training program for general middle-aged and older adults should be 2 to 3 days per week for large muscle group with an interval of at least 48 hours for the same muscle group; each muscle group should be trained in 2 to 4 groups with 8 to 12 repeats for each group and 2 to 3 minutes rest between groups.^[35]^

In this study, the grip strength of upper limb muscles, leg lifts that reflected the strength of trunk muscles, and 30-s repeated sit-to-stand actions that reflected the strength of lower limb muscles of older adults in the control group became worse significantly, which showed without training, the muscle strength of older adults decreased with age; whereas, the strength of upper limb, trunk, and lower limb muscles of older adults in the training groups were significantly improved; the longer the training time was, the more obvious the effect was. These findings showed that the resistance exercise combined with elastic ball and elastic band could effectively improve the muscle strength of the bedridden older adults, and the effect would be better as time went on, which was consistent with the research results in China and abroad. A study on 6-month elastic band exercise in the older adults showed that the elastic band exercise could improve their physical function and muscle quality.^[9]^ Qi^[36]^ trained the healthy older adults for 3 months using the elastic band progressive training method, and she found that the body shape and body function indexes of the older adults were significantly improved after training. Several studies showed that resistance training could exercise upper limb muscles, abdominal muscles, back muscles and lower limb muscles, effectively strengthen the strength of the subject’s shoulders, upper arms and lower limbs, and play an important role in training the waist, abdomen and back, which could enhance joint muscle strength, strengthen joint stability, and help to improve the static balance ability and quality of life.^[37,38]^ Therefore, the resistance exercise combined with elastic band and elastic ball is an effective intervention method to improve the physical function and increase muscle strength of the older adults.

The results of this study showed that the vital capacity of patients was significantly improved after training, while in the control group, the vital capacity decreased than before, which was consistent with the results of other domestic and foreign studies. Kanegusuku et al,^[39]^ Chen et al,^[40]^ and Lian Tang et al^[41]^ all showed that a certain intensity of resistance exercise could also improve the lung function of older adults. This finding suggested that the cardiopulmonary function of the older adults declined with the advancing age and circulation resistance exercise was a progressive resistance training method, which could improve the cardiovascular function.^[42]^

In this study, the blood pressure of the older adults was not significantly improved. This result needed to be further verified as it was different from some of the research results obtained in China and abroad. Foreign study found that resistance exercise also played a positive role in the prevention and control of hypertension.^[43]^ It has been found that the systolic blood pressure of the older adults decreased after resistance exercise training, but there was no significant difference, while the diastolic blood pressure increased.^[23]^ The reason for this finding might be that the blood pressure of the older adults is affected by many factors, such as their basic diseases, medications, measurement time, sleep quality, emotion, and other activities. In this study, most of the participants were older adults who attained advanced age and had poor basic physical conditions. Most of these patients had high blood pressure as their sleep quality, emotion, and other activities resulted in poorly controlled blood pressure and fluctuations. These could be the reason that the effect of blood pressure improvement after training was not satisfactory.

Although the RHR and the LVEF were not statistically different between the groups in this study, the LVEF of patients in the training group was better at 6 months than pre-training and if the training time was to be extended, the effect would be more obvious. The reason for this finding might be the different exercise methods and intervention time led to different results. Du et al^[8]^ compared elastic band resistance exercise with moderate-intensity aerobic exercise, and found that the cardiac function of elastic band group was significantly better than that of aerobic exercise group after 1 year of intervention. But Redwine et al^[44]^ studied the patients with heart failure (mean age = 66 years, range = 45–89 years) who were randomized to 16-week of tai chi, resistance band exercise, or treatment as usual, and found no group differences occurred over time in end-systolic volume and LVEF. Li et al^[45]^ applied the low-intensity, home-based exercise protocol for 12 weeks in the older adults over 75 years with CHF and there were also no significant improvements in RHR and LVEF. Some studies showed that resistance exercise combined with aerobic exercise could effectively improve the left ventricular ejection fraction and the effect was better than only by the resistance exercise.^[46,47]^ In this study, only by the low-intensity resistance exercise for 6 months, the changes in the heart rate and LVEF were small, which suggested that extending the training time or using the aerobic combined resistance exercise would be more obvious.

The results of this study showed that the levels of HDL in the training groups were significantly improved after resistance exercise, and the effect was better with the extension of training time. The level of triglyceride decreased with time going on in the training group, but there was no significant difference between the 2 groups. This suggests that the elastic band resistance exercise could improve the blood lipid metabolism of the older adults to a certain extent. According to the research by American College of Sports Medicine, resistance exercise could increase HDL by 8% to 12% and reduce LDL by 13% to 23% and triglyceride by 11% to 18%.^[48]^ Hou^[23]^ applied a 12-week low-intensity circulation resistance exercise on the older adults and found that after intervention, the levels of HDL increased and that of LDL decreased, but triglyceride and total cholesterol values changed a little in the training group, while triglyceride, total cholesterol, and LDL in the control group increased and HDL decreased. Codella et al^[49]^ found that resistance exercise with exercise time ≥ 12 weeks and intensity of 60% to 80% 1RM significantly improved the blood lipid metabolism of patients, but the resistance movement mode in their study was with the help of sports equipment, so this conclusion could not be extended to other resistance training, such as elastic band exercise. The reason for different effects may be that different exercise modes, frequencies, intensity and time of exercise could cause different effects. Resistance training in different forms, frequencies, intensity and time has different total energy consumption for the older adults, and the change of blood lipid mainly depends on the total energy consumption.^[50]^ Therefore, increasing frequency, time and intensity of the resistance exercise will significantly improve the blood lipid. The participants of this study were bedridden older adults, and we used low-load and low-intensity resistance exercise. The impact on the basic metabolism of the older adults was also low, leading to the results not as significant as those in other similar studies.

In terms of blood glucose metabolism, there was no significant statistical difference in blood glucose levels between the 2 groups, which was inconsistent with other studies. A foreign study found that the elastic band training applied for 60 minutes, 3 times a week during 12 weeks within 12 to 15 (HRR 60–80%) of Borg’s rating scales of perceived exertion (RPE, 15-point scale) had a positive effect on improving glycosylated hemoglobin levels and insulin resistance in older adults with diabetes.^[51]^ Mavros et al^[52]^ also proved that resistance exercise can improve insulin resistance, control blood glucose and reduce glycosylated hemoglobin. The reason may be that different exercise intensity could cause different results. The meta-analysis results of Lee et al^[53]^ showed that the effect of high-intensity resistance training on improving blood glucose for the older adults was better than that of low-intensity training. Besides, different exercise methods could also cause different results. Many studies showed that aerobic combined resistance exercise had the best effect on improving insulin resistance and controlling blood glucose.^[54,55]^ In this study, only low-load and low-intensity within 10 to 11 (30–50% of maximum load, HR 105–110 beats/min) of Borg’s rating scales of perceived exertion (RPE, 15-point scale) resistance exercise was applied for 55 minutes, 3 times a week during 6 months, and the effect of controlling blood glucose was not significant. These findings suggested that increasing the intensity of resistance exercise or combined aerobic exercise could plays a positive role in blood glucose control in the older adults.

There are some limitations in this study. Because the sample size is small and the evidence provided is limited, a multi-center and large sample size population is recommended for further verification. Although the number of bedridden older adults is increasing with advancing age, there are more older adults who can take part in out of bed activities or outdoor activities. It is necessary to design some resistance exercise movements for them to improve muscle strength and physical condition. In this study, certain exercise movements were not suitable for sitting and standing, so it was suggested that sitting and standing movements were designed to meet the activity needs of these older adults. For safety and conservation reasons, only low-load and low-intensity resistance exercise was selected in this study and the effect of some indicators was not significant. The American Heart Association and the American Sports Medical Association recommend that resistance exercise for the older adults over 65 years old is mainly progressive weight-bearing exercise with medium or large intensity.^[21]^ So the parameters should be evaluated with moderate-intensity exercise and extension of the intervention time in future studies, so that the long-term training effects of exercise under different load intensities could also be investigated further.

## 5. Conclusion

In conclusion, low-load and low-intensity resistance exercise using elastic band and elastic ball is a simple, safe, and effective exercise method for the bedridden older adults. This type of exercise may improve the physical function and muscle strength of the older adults, and effectively improve the lung function and blood lipid metabolism to a certain extent, and older adults also have better compliance and safety, which makes it an important supplement of exercise rehabilitation of the older adults in China.

## Acknowledgments

We are grateful to Hai-yan Qiu, Lin Bo, Wei Liu, Hong-di DU, Sha Nan, Department of Health Care, Peking Union Medical College Hospital, assisted with data collection. These contributors were paid for their roles. I also expressed my gratitude to the staff, residents, caregivers and the parents who participated in our study in Geriatric ward, Neurology ward and Physiotherapy department.

## Author contributions

**Writing – original draft:** Yingjie Wang, Xiaopeng Huo, Xiaojing Wang, Hongwei Zhu, Xiaoxing Lai, Tong Yu.
